# Energy Absorbing Properties Analysis of Layers Structure of Titanium Alloy Ti6Al4V during Dynamic Impact Loading Tests

**DOI:** 10.3390/ma14237209

**Published:** 2021-11-26

**Authors:** Dominik Głowacki, Wojciech Moćko, Michał Marczak, Anna Głowacka, Cezary Kraśkiewicz

**Affiliations:** 1 Institute of Aeronautics and Applied Mechanics, Faculty of Power and Aeronautical Engineering, Warsaw University of Technology, Nowowiejska 24, 00-665 Warsaw, Poland; dominik.glowacki@pw.edu.pl; 2 Faculty of Engineering and Economic, Ignacy Mościcki’s University of Applied Sciences in Ciechanów, Narutowicza 9, 06-400 Ciechanów, Poland; w.mocko@electricboats.pl; 3 Institute of Manufacturing Technology, Faculty of Mechanical and Industrial Engineering, Warsaw University of Technology, Narbutta 85, 02-524 Warsaw, Poland; 4 Institute of Machine Design Fundamentals, Faculty of Automotive and Construction Machinery Engineering, Warsaw University of Technology, Narbutta 84, 02-524 Warsaw, Poland; anna.glowacka@pw.edu.pl; 5 Institute of Roads and Bridges, Faculty of Civil Engineering, Warsaw University of Technology, Al. Armii Ludowej 16, 00-637 Warsaw, Poland; c.kraskiewicz@il.pw.edu.pl

**Keywords:** fracture toughness, dynamic impact test, finite element analysis

## Abstract

This paper presents the testing methodology of specimens made of layers of titanium alloy Ti6Al4V in dynamic impact loading conditions. Tests were carried out using a drop-weight impact tower. The test methodology allowed us to record parameters as displacement or force. Based on recorded data, force and absorbed energy curves during plastic deformation and sheet perforation were created. The characteristics of the fractures were also analyzed. The impact test simulation was carried out in the ABAQUS/Explicit environment. Results for one, two, and three layers of titanium alloy were compared. The increase in force required to initialize the damage and the absorbed energy during plastic deformation can be observed with an increase in the number of layers. The increase in absorbed energy is close to linear. In the simulation process, parameters such as Huber–Mises–Hencky stress value, equivalent plastic strain, temperature increase, and stress triaxiality were analyzed.

## 1. Introduction

Titanium and its alloys are common materials that have been used since the 1960s. Their desirable properties, which include low density, high mechanical strength, heat resistance, low-temperature fracture toughness, low thermal expansion, resistance to in-service corrosion processes, and biocompatibility, allow for wide applications in biomedical, automotive, aviation, and military engineering. An example is the modern method of joining parts using the electromagnetic riveting process (EMR), a high-speed impact connection technology with the advantages of fast loading speed, large impact force, and stable rivet deformation [1]. However, a high friction coefficient, poor resistance to abrasive wear, and a low hardness limit the possible areas of application of titanium alloys. Therefore, the proper use of these materials requires knowledge of their characteristics and properties [2,3,4].

There are many publications on the behavior of titanium alloys under static loading [5,6]. However, dynamic loading has not been sufficiently defined. Some papers concerning the high strain rate properties of titanium alloys are available [7,8,9,10], but much work must still be undertaken, especially in the areas of experimental techniques, microstructural analysis, and modeling, to fully define the constitutive properties of these materials. Invoking a variable load over a short time period can cause a change in the behavior of titanium alloys [11]. In some areas, such as aviation and the military, the level of dissipation or absorption of impact energy in a material is also important. However, detailed information on this, such as the energy absorption level related to mass, is often absent [12].

The aim of this study was to determine the energy-absorbing properties of layered structures of titanium Ti6Al4V alloy under dynamic impact conditions using a drop-weight impact tester, as well as to determine the basic material properties for high strain rate deformation for numerical simulation. The analysis allowed us to estimate the energy required to break a single sheet and the layered structure sheets to determine the puncture characteristics and the influence of layered structures on the amount of energy required.

A visual assessment of the mechanism of developing damage and absorbed energy value during experimentation depending on the number of layers was performed. In addition, a force value analysis of failure initiation was performed. For this purpose, appropriate sheet metal specimens of 1 mm thickness were prepared to perform dynamic impact tests on a drop-weight tester. The mechanical and physical properties of titanium alloy Ti6Al4V have been broadly described in the literature. These alloys can be widely applied in many fields, such as 3D printing of ultralight sandwich structures with corrugated cores in aerospace [13] and medical [14] applications.

The innovative approach in this paper is based on a multi-layer analysis of energy absorption by layered structures of titanium alloy during dynamic impact loading tests. This paper presents experimental data together with the numerical solution of a given impact load. The multi-layer structures are more effective in terms of strength and energy absorption compared to monolithic layers.

Using a drop-weight impact method allowed us to determine the energy-absorbing properties of a Ti6Al4V titanium alloy sheet. Further research could be used to analyze the behavior of polymer-titanium and polymer-glass-titanium composites.

## 2. Materials and Methods

### 2.1. Properties and Structure of the Titanium Alloy Ti6Al4V

**Figure 1:**
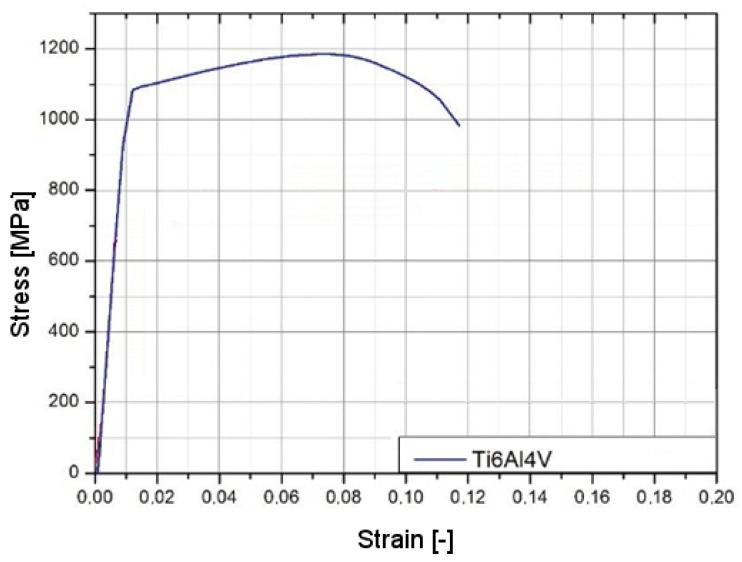
Stress–strain quasi-static characteristic for Ti6Al4V [22].

Titanium Ti6Al4V is a dual-phase alloy composed of *α* and *β* phases. Stabilization of the phases corresponds to the principal alloying elements, aluminum stabilizing alpha, and vanadium beta phase [15,16,17]. Chemical compositions of the Ti6Al4V were used according to ISO 5832/3 [18]. The selected titanium alloy exhibits very good mechanical properties [19], which is why Ti6Al4V is commonly used in technology, especially in the aerospace industry. Depending on the manufacturing process, titanium alloys are characterized by anisotropy, low density, a Young’s modulus of 115 GPa (comparable to steel: 205–210 GPa), high strength with good ductility, and a high melting point. The main mechanical properties are presented in Table 1 and Figure 1. Another characteristic advantage of titanium alloys is corrosion resistance at ambient temperatures, e.g., in the air or an industrial atmosphere. Compared to pure titanium, Ti6Al4V alloy has greater strength with the same stiffness and thermal properties [20]. However, Ti6Al4V also has the notable disadvantage of easily reacting with other materials at higher temperatures. This entails a number of obstacles, such as the need for using unconventional methods in the melting and casting processes, which leads to an increase in production and processing costs.

The chemical and physical properties that might occur during a given process type in Ti6Al4V titanium alloy processing must be considered due to chemical reactivity, low modulus of elasticity, and high strength. Ti6Al4V can be submitted to processes including casting, welding, cutting, and plastic processing. The variety of manufacturing methods and process parameters significantly affects the anisotropy of the material and its mechanical properties, which should always be considered in the design process.

### 2.2. Specimens

**Figure 2:**
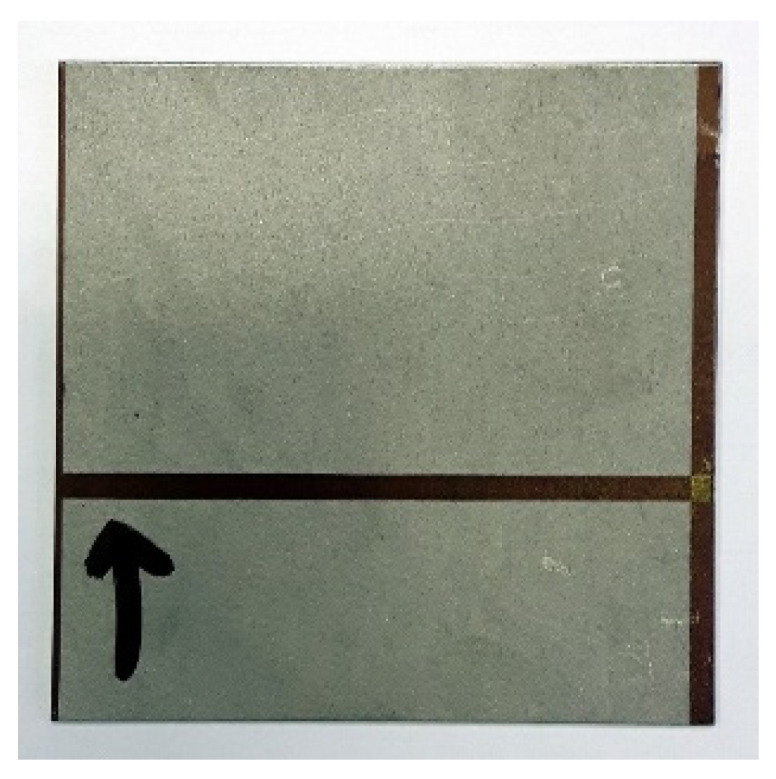
Plate made from Ti6Al4V.

Specimens were made from a cold-rolled metal sheet of Ti6Al4V of 1 mm thickness. For the experiments, 18 plates of 80 mm × 80 mm were cut from the sheet. Each specimen was described and marked with an arrow showing the rolling direction, according to the anisotropic material properties. A specimen is shown in Figure 2.

Non-standard impact tests were used. There is no possibility to measure the direct impact strength on a sheet of 1 mm thickness, but it is possible to determine the energy absorbed by the puncture process. In this case, the state of the stress test changed to two-dimensional.

### 2.3. Drop-Weight Impact Tester 

**Figure 3:**
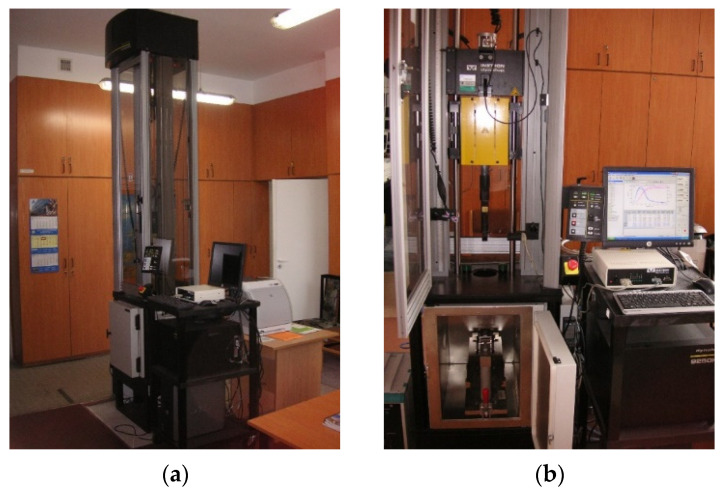
Post view: (**a**) side; (**b**) front.

Dynamic impact tests were carried out on a drop-weight impact tester INSTRON with Dynatup 9250 HV (Instron, Norwood, MA, USA) column equipped with an Impulse control and data processing system (Figure 3). The INSTRON machine offers 20 m/s speed (forced drive), simulation of discharge height of 20.4 m, and impact energy from 4.5 to 1600 J. The use of this post enabled the impact test to record parameters such as impact velocity, energy change, load, yield strength, deformation, and time.

**Figure 4:**
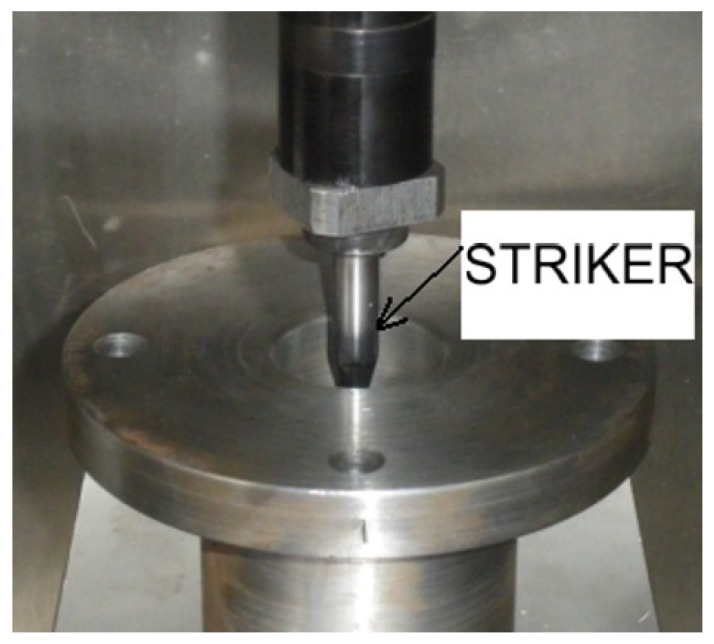
Striker used in impact tests.

The striker is made of NC3 tool steel with a hardness of 48 HRC (Figure 4). It has a diameter of 10 mm, a cone-shaped blade with a tip angle of 30° ± 1°, and a radius of curvature of 2 mm. The striker is shown in Figure 4. It is easily seen that, apart from the cylindrical shape of the striker, the geometric dimensions fit Charpy’s standards.

The recorded results of impact tests were graphs of absorbed energy (impact work) (J) and load (kN) as a function of the recorded displacement (mm) of the specimen material and in the form of the numerical values, which were recorded automatically. The arrays for individual trials collected the values of the most important parameters measured and recorded during the tests by the Impulse control and the data processing system used.

### 2.4. Calibration of Johnson–Cook Model for FE Simulation

The Johnson–Cook (JC) model was calibrated for the simulation because a reliable material model was required for numerical simulation at high deformation rates.

FEM analysis was performed using the ABAQUS/Explicit environment (ABAQUS Inc. Providence, MA, USA). The Johnson–Cook model [23,24] was used for the simulation. The JC is a constitutive model describing the plastic behavior of the material at high strain, strain rate, and temperature. The JC Equation (1) describes the empirical stress relationship of the Huber–Mises–Hencky (HMH) equation.

(1) $$\sigma =\left(A+B\varepsilon^{n}\right)*\left[\;1+C*\ln\left(\frac{\dot{\varepsilon }}{\dot{\varepsilon_{0}}}\right)\right]*\left[1-Q^{m}\right]$$

where $Q=\frac{T-T_{0}}{T_{melt}-T_{0}}$.

There are different methods of calibrating the JC model. The method used in this study was calibrated according to the commonly used scheme: determination of constants (*A*, *B*, and *n*) based on isothermal tensile (or torsion) tests; determination of *C* constant under quasi-static or dynamic conditions at different deformation rates; and *m* coefficient in dynamic conditions for different temperature values (in this case, due to no possibility of testing under different temperature conditions, the *m* coefficient was selected from the literature [25]).

Due to the obtained mechanical characteristics of the titanium alloy, it was possible to calibrate the Johnson–Cook model as presented in Equation (2).

**Figure 5:**
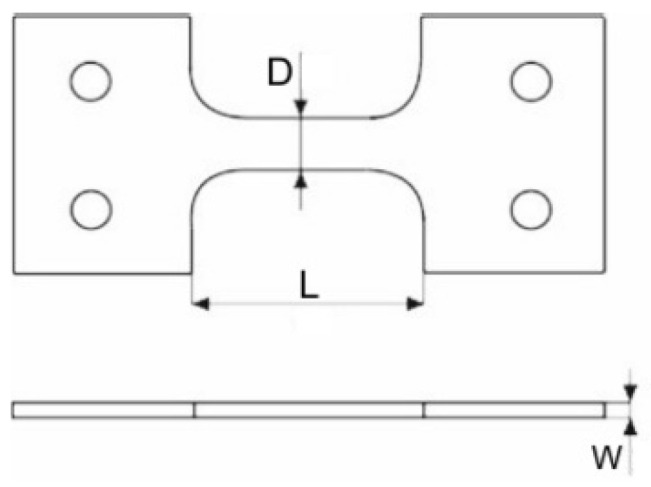
Specimen scheme used in experiments on Kolsky bar (*L*—gauge length).

**Figure 6:**
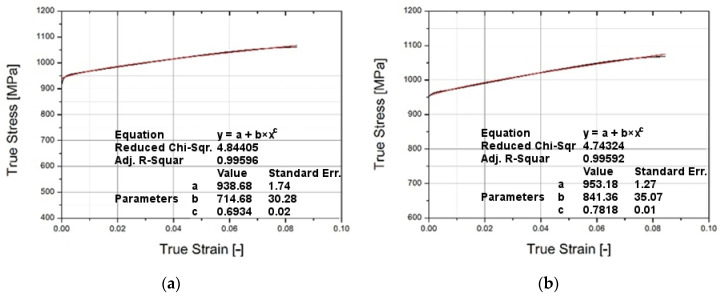
Stress–strain characteristic for specimen’s gauge length: 18 mm (**a**) and 30 mm (**b**) with power function approximation.

**Figure 7:**
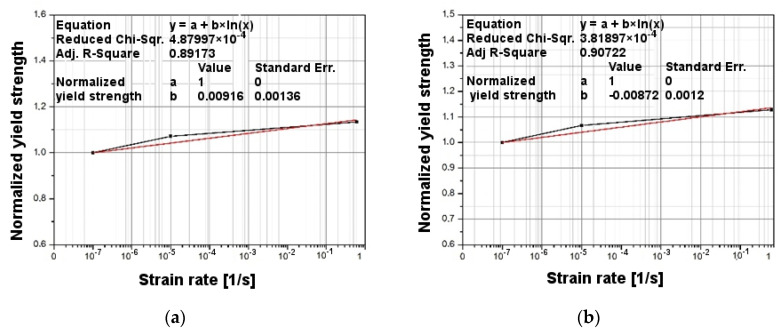
Normalized yield strength characteristic at strain value ε = 0.02 for specimen gauge length: 18 mm (**a**) and 30 mm (**b**).

Calibration was made for two cases: for specimens with a gauge length (*L*) of 18 mm, and for specimens with a gauge length of 30 mm (Figure 5). In the first step to determine the coefficients *A*, *B*, and *n* for the strain rate 0.0001/s, the stress–strain characteristic curve was approximated by the power function. The results are shown in Figure 6. The results shown in Figure 6 for strain value *ε* = 0.02 were recorded for three different strain velocities. Figure 7 shows the results for two specimens (18 and 30 mm), where the normalized yield strength is shown on the vertical axis. The value of the coefficient *m* was chosen based on the literature [25]. The values of the Johnson–Cook coefficients are set forth in Table 2. The real stress–strain characteristics were derived from the engineering values obtained in the tensile tests using the logarithmic function in Equations (2) and (3).

(2) $$\varepsilon_{T}=\ln\left(1+\varepsilon \right)$$

(3) $$\sigma_{T}=\sigma \left(1+\varepsilon \right)$$

**Figure 8:**
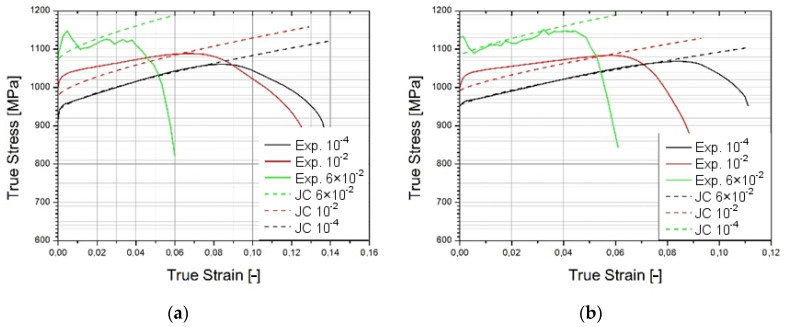
Stress–strain characteristics for 3 strain rates compared to Johnson–Cook results for specimen gauge length of 18 mm (**a**) and 30 mm (**b**).

Based on the obtained results, stress–strain characteristics for 18 mm and 30 mm gauge lengths were developed for three different strain rates. Using Johnson–Cook coefficients, similar value characteristics for this model were obtained. The comparative characteristics are presented in Figure 8. It is easy to observe that as the strain rate increases, the difference between real and computational values rises. However, received values are within the limits of acceptability.

The remaining material data used in the simulation were: density 4510 kg/m^3^; Young’s modulus 115 GPa; Poisson ratio 0.31; Taylor–Quinney coefficient 0.9; specific heat 523 J/(kg∙K).

To verify the correctness of our methodology for determining the energy-absorbing properties of titanium alloy, numerical simulations of the sheet metal impact test were made using the above material models.

**Figure 9:**
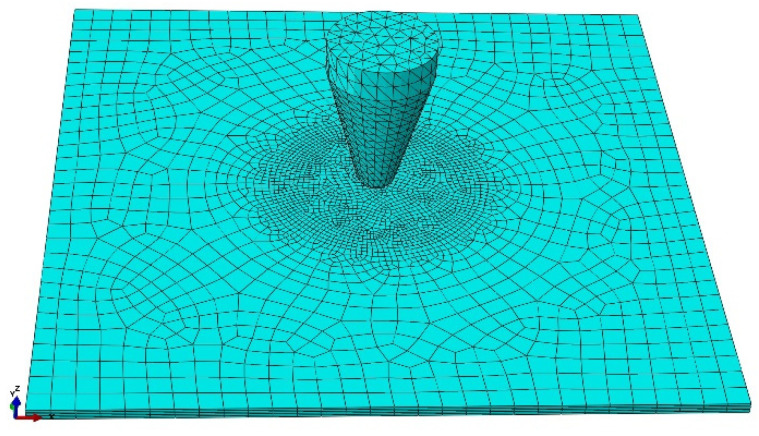
Mesh model of a single plate.

The model of sheet metal and mesh is shown in Figure 9. The sheet metal model was divided into 14,995 finite elements (element type: C3D8R) for the analysis. The mesh was compacted at the puncture area due to high displacement values and to precisely visualize the material deformation. The striker was modeled as a rigid body for simulation purposes. The plate was fixed at its edges and the striker was given a starting value of 12.3 m/s, which corresponds to the value measured during the experiment. A friction interaction of 0.1 was introduced between the surface of the striker and the mesh of the plate. The value of the friction coefficient was determined based on the successive simulations to achieve results most closely related to the experimental values.

## 3. Results and Discussion

During experiments, nine dynamic impact tests were carried out at room temperature (296.15 K). A total of three attempts were made for the individual case to obtain statistical data. Specimens were punctured separately (specimens 1, 2, and 3); specimens from 4–9 were layered in pairs (4–5, 6–7, and 8–9); specimens from 10–18 were layered in threes (10–11–12, 13–14–15, and 16–17–18).

**Figure 10:**
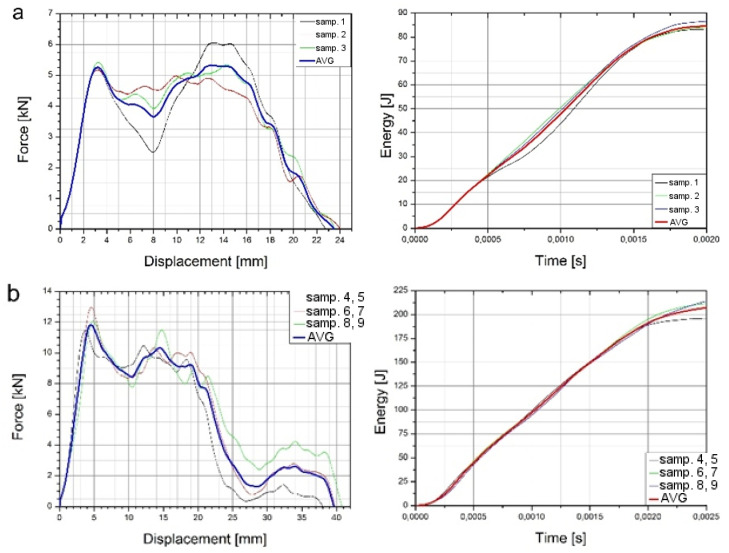

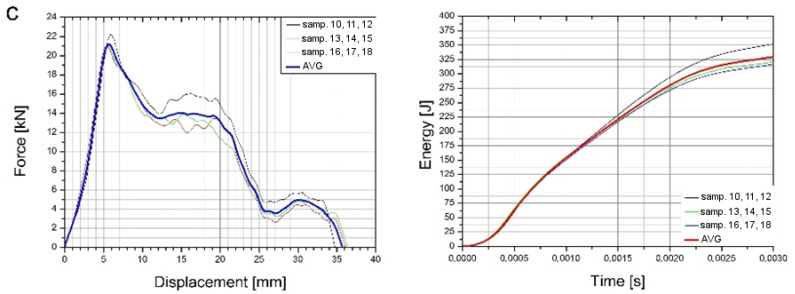
Force–displacement relations during puncture and energy during puncture charts for 1 plate (**a**), 2 plates (**b**), and 3 plates (**c**).

**Figure 11:**
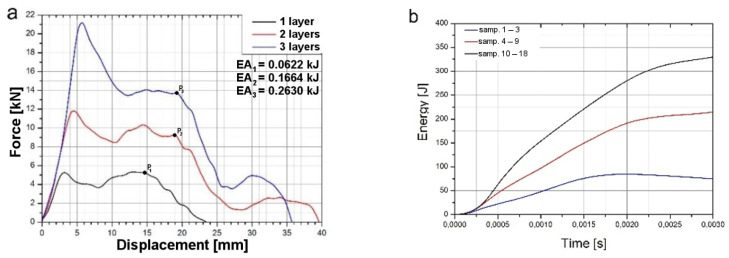
Comparison of averages: force–displacement (**a**) and energy during puncture (**b**) graphs.

**Figure 12:**
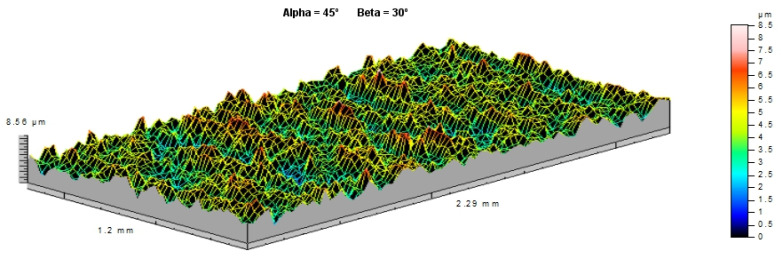
Roughness of the samples *Sa* = 0.833 µm, *Sq* = 1.05 µm, *St* = 8.56 µm, *Str* = 0.262 µm, and *Sk* = 2.1 µm (Taylor Hobson Ltd., Leicester, U.K.).

**Figure 13:**
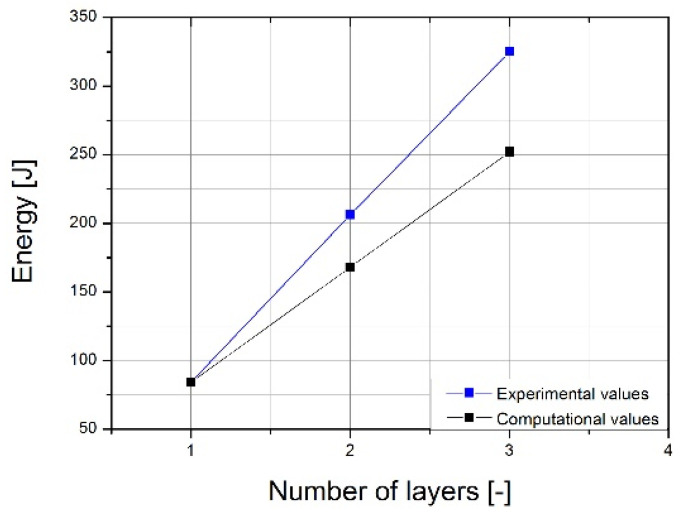
The dependence of energy during puncture on the number of layers of the sheet for the value measured during the experiment and the calculation value.

As a result of the study, graphs of force during puncture in the displacement function, as well as energy during puncture, were obtained. The results for individual cases shown in Figure 10 and averaged in Figure 11 are also a comparison of results for each case, as can be observed. It clearly shows an increase in strength during puncture relative to the number of layers. The energy needed to puncture double-plate (205 J) is greater than twice the energy required to puncture a single plate (84 J × 2 = 168 J). Similarly, three-layer structure energy (325 J) is greater than three times the energy required for a single plate (3 × 84 J = 252 J). That might be due to the fact that the striker energy is dispersed not only by the deformation of the material and the energy required to destroy it but also by friction between each layer. The coefficient of friction between each layer is correlated with the roughness of the samples, as shown in Figure 12. A comparison of the value obtained from the experiment and the energy value calculated based on a single-sheet puncture is shown in Figure 13. It is easy to see that this relationship shows characteristics similar to a linear one. The study of sheet metal multi-layer structures, consisting of sheet metal packaging, can be an introduction to the analysis of polymer-titanium and polymer-glass-titanium composites’ behavior.

**Figure 14:**
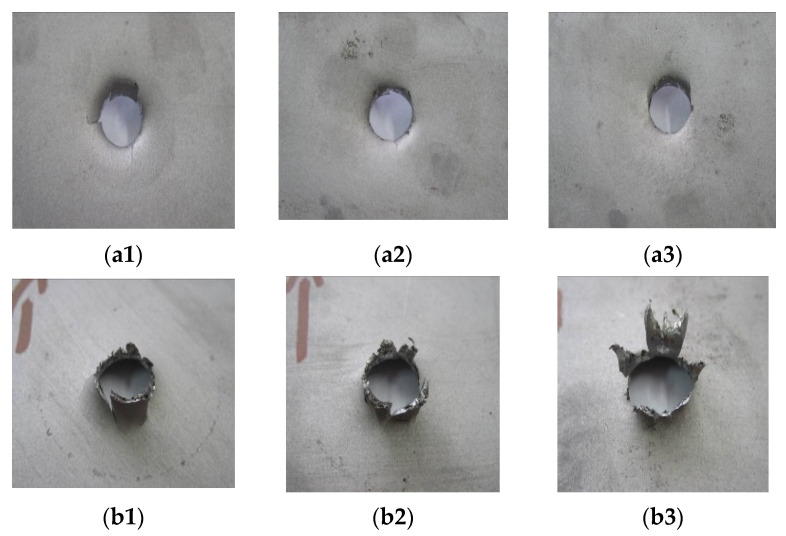
Results of dynamic impact tests of 1 mm Ti6Al4V sheet: (**a1**–**a3**) view from above; (**b1**–**b3**) view from bottom.

**Figure 15:**
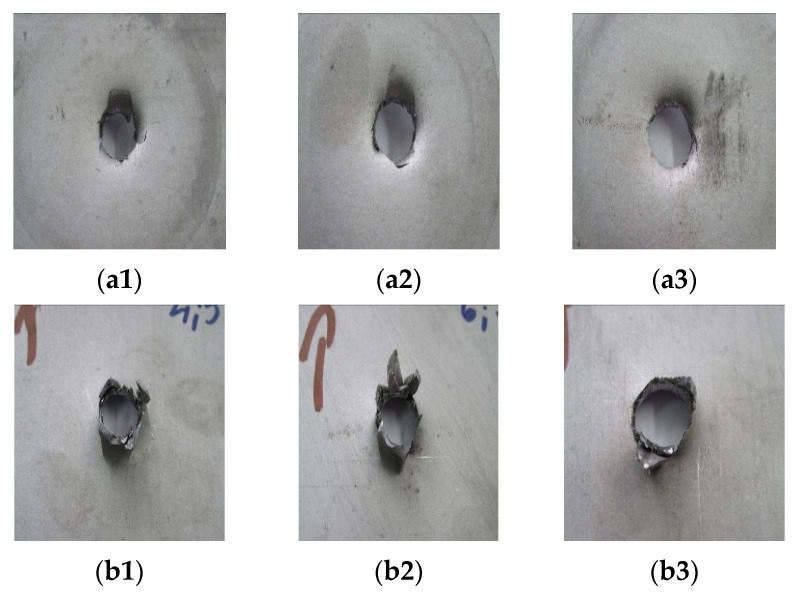
Results of dynamic impact tests of 1 mm Ti6Al4V sheet in layer structure of pairs: (**a1**–**a3**) view from above; (**b1**–**b3**) view from bottom.

**Figure 16:**
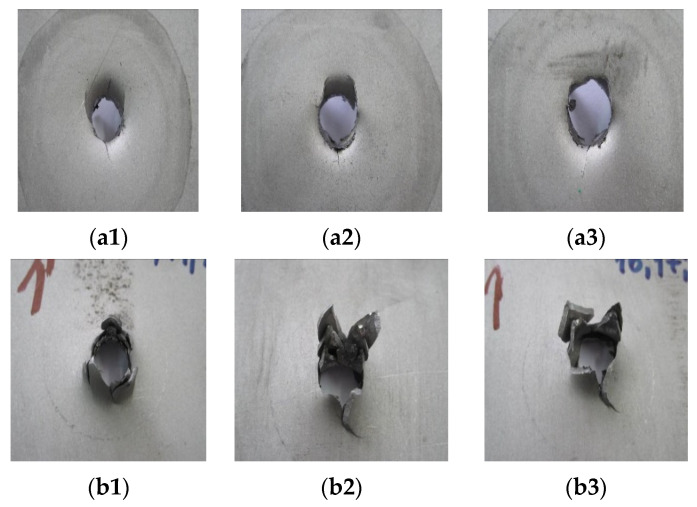
Results of dynamic impact tests of 1 mm Ti6Al4V sheet in layer structure of threes: (**a1**–**a3**) view from above; (**b1**–**b3**) view from bottom.

Figure 14, Figure 15 and Figure 16 show the results of the impact tests with a comparison to the finite element simulation. The form of material failure (bursting) has a petaling character. In titanium structure sheet tests, the first layers are most crushed, grated by contact between the plate and the striker. The last layers show the greatest deformation.

### 3.1. Calculation of Specific Energy Absorption W_EA_

The energy-consuming structures used in the aerospace industry should be of the lowest possible weight, but also of high strength and stiffness to specific mass ratio. One of the parameters describing the energy consumption is the total impact energy absorption *EA* (kJ), equivalent to the area under the load–displacement curve calculated up to points *P*_1_, *P*_2_, and *P*_3_ of the sudden curve fall (Figure 11a).

In order for energy-absorbing structures to best absorb the impact energy, the destruction of these structures must not be carried out in an abrupt manner, as in beam damage during global buckling. Instead, the destruction should be carried out progressively, so that each volume of the specimen is crushed (destroyed) into the smallest particles.

The process of progressive destruction depends mainly on the mechanical properties of the material, its structure, shape, and the geometry of the specimens.

The specific energy absorption capability (4) *W_EA_* of a composite material is defined as the energy absorbed per unit mass of material as

(4) $$W_{EA}=\frac{EA}{m}\left[\frac{kJ}{kg}\right]$$

where *m* is the mass of the composite material.

**Figure 17:**
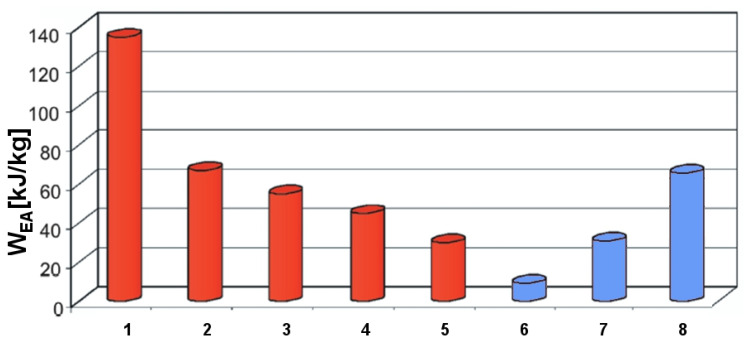
*W_EA_* depending on material type: 1—carbon/polyetheretherketone (PEEK), 2—carbon/epoxy, 3—glass/epoxy, 4—short glass fibers/epoxy, 5—steel, 6—titanium (single layer), 7—titanium (double layer), 8—titanium (triple layer) [26].

Examples of the values for the highest relative absorption energy *W_EA_* for several types of materials are given in Figure 17.

### 3.2. FE Simulation Results

**Figure 18:**
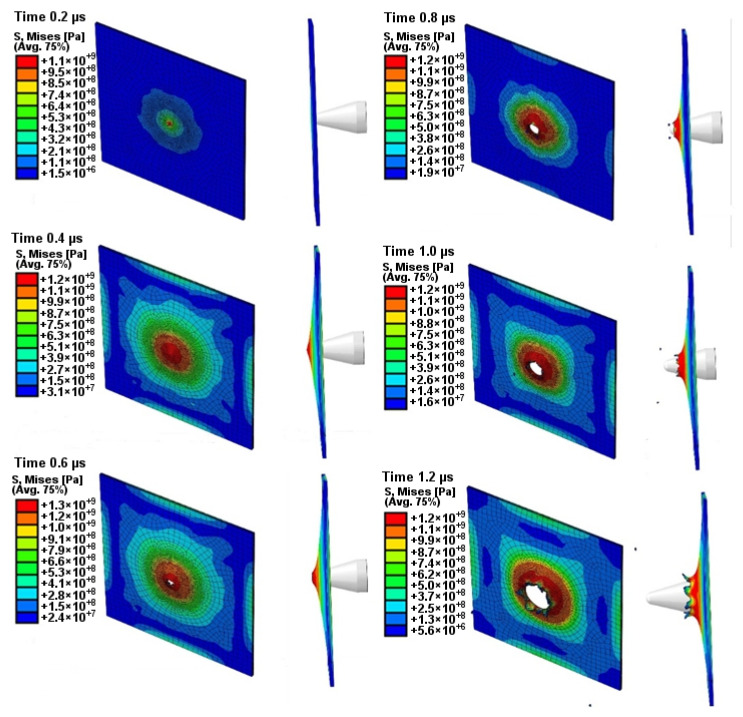
HMH stress visualization of one titanium alloy plate during the impact test.

Simulations were created to develop a material model of Ti6Al4V matched to high strain rate tests, to compare the results with those of the experiments, and to define properties that could not be tested by experiments such as temperature and stress state during puncture. The Johnson–Cook coefficients in Table 2 were used in the simulation. The Huber–Mises–Hencky stress characteristic (HMH) during the puncture process was obtained in the simulation, as shown in Figure 18. As a result of the simulation, the maximum stress was approximately 1300 MPa, which can be considered to be in line with experimental data.

**Figure 19:**
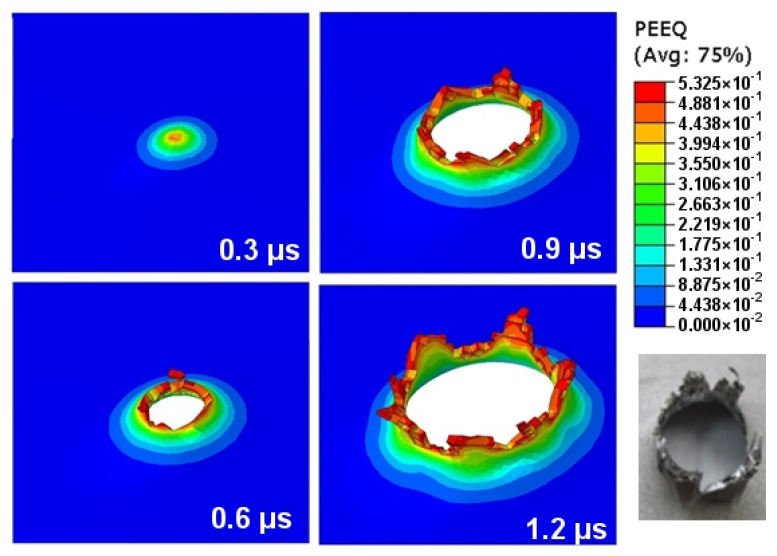
PEEQ strain visualization during the puncture of a sheet made of titanium alloy.

Material crumbling and the formation of characteristic flakes comparable to the experiment can be observed during damage propagation. The highest value of reduced stress (1.3 × 10^9^ Pa) was recorded for a step of 0.6 µs. At this point, the material undergoes progressive decohesion. At the next step (*t* = 0.8 µs), spring relief of the material can be observed by comparing the stress areas. At the time of decohesion, reduced stress greater than or equal to 2.8 × 10^8^ Pa is present across the majority of the specimen area. However, in the next step (*t* = 0.8 µs), the reduced stress importantly decreases over a significant area below the value of 1.4 × 10^8^ Pa, only to increase again in the next simulation step (*t* = 1.0 µs). This increase continues until the punch fully passes through the specimen. Figure 19 shows the strain visualization of equivalent plastic strain (PEEQ) during sheet metal puncturing. The maximum strain value was 0.532. This value is greater than that available in the literature [27].

**Figure 20:**
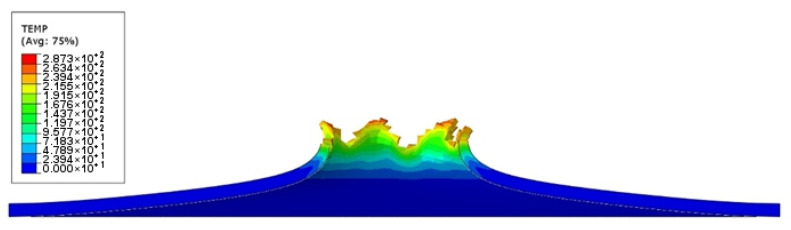
Temperature increase visualization due to the puncture of the titanium alloy sheet by the striker—section view.

**Figure 21:**
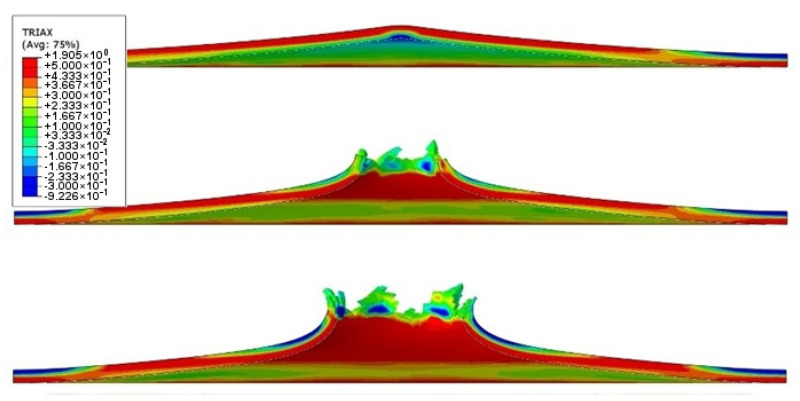
Stress triaxiality visualization in section view during puncture.

In the ABAQUS simulation process, tests such as temperature increase, stress triaxiality, and force at failure initiation were carried out. Figure 20 shows the sheet metal temperature increase at the puncture area where the maximum increase from the initial temperature was 287.3 K. Taking into account the initial conditions (initial temperature 296.15 K) the sheet was heated to 583.45 K. Figure 21 shows the stress during the puncture of the specimen. Compressive stress occurred when there was tensile stress on the opposite side of the sheet at the point where the striker contacted the sheet. When the sheet is bent by puncturing, at the point of contact with the striker the compressive stress undergoes tensile stress, and the stress changes into compression with the development of the deformation. The maximum triaxiality stress value is 1.9, which is higher than that in the literature [27].

**Figure 22:**
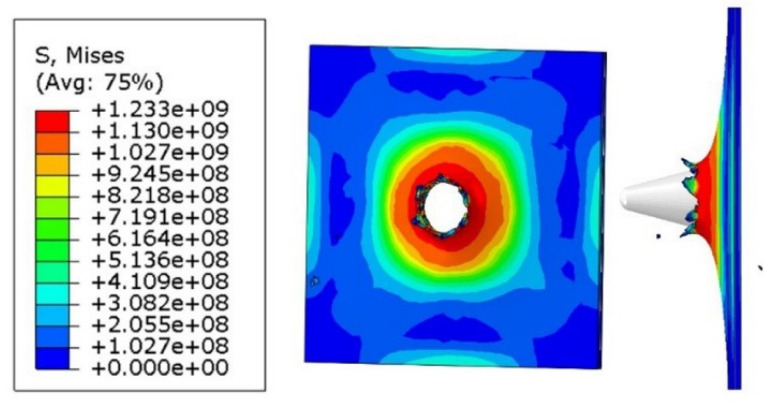
HMH stress visualization for two layers of titanium alloy.

**Figure 23:**
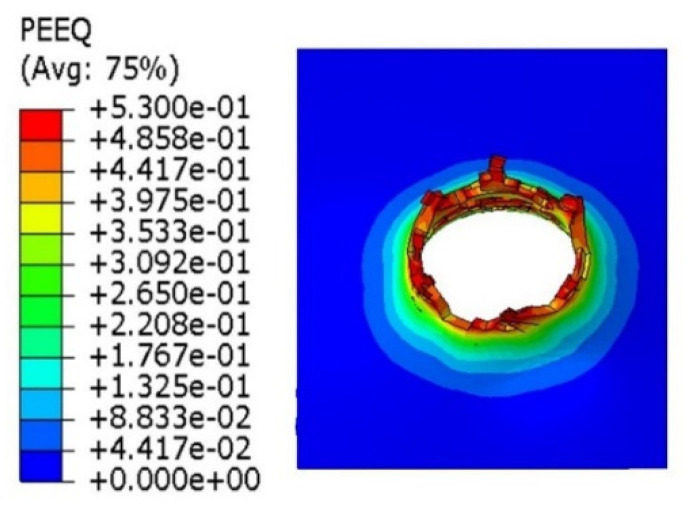
PEEQ visualization for two layers of titanium alloy.

**Figure 24:**
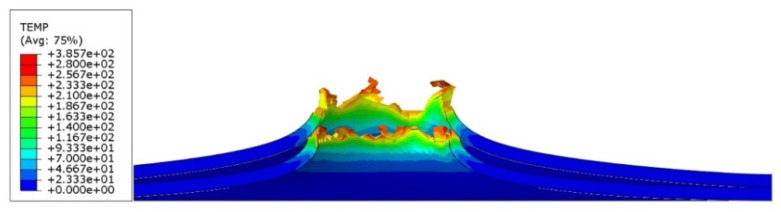
Temperature increment visualization due to the puncture of two layers of titanium alloy sheet by striker—section view.

**Figure 25:**
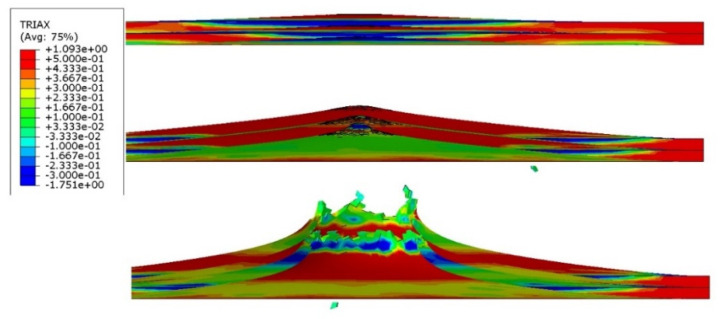
Stress triaxiality visualization in section view during the puncture of two titanium alloy sheets.

As part of the ABAQUS simulation for two layers of sheet metal, analogous calculations and visualizations were performed as in the case of a single layer. Visualizations of stress and strain are shown in Figure 22 and Figure 23. The maximum values of the parameters are the reduced HMH stress of 1233 MPa and PEEQ strain of 0.53. The visualization of the temperature increase at the puncture point in Figure 24 indicates a temperature increase of 385 K. This is 99 K more than for a single layer. Such a difference may be due to the interaction between the surfaces. The nature of the stress triaxiality condition can be considered analogous to the first simulation (Figure 25).

**Figure 26:**
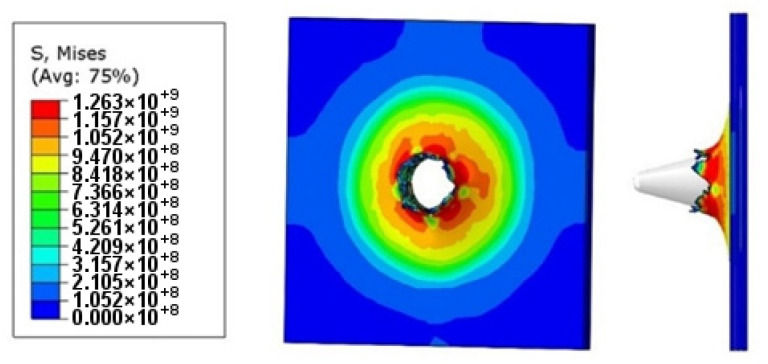
HMH stress visualization for three layers of titanium alloy.

**Figure 27:**
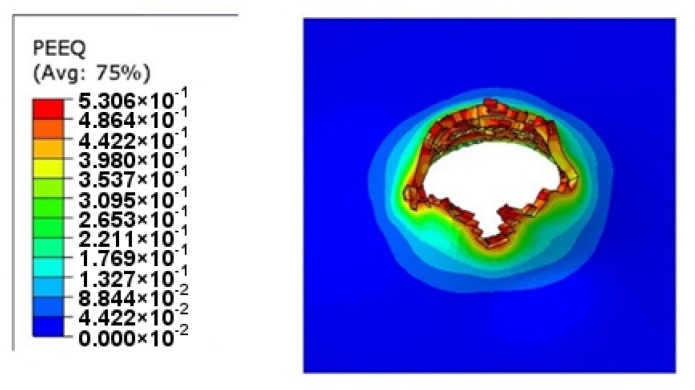
PEEQ visualization for three layers of titanium alloy.

**Figure 28:**
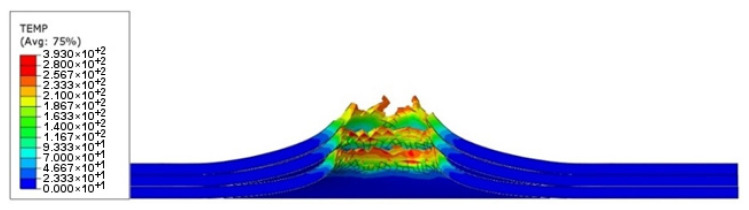
Temperature increment visualization due to the puncture of three layers of titanium alloy sheet by striker—section view.

**Figure 29:**
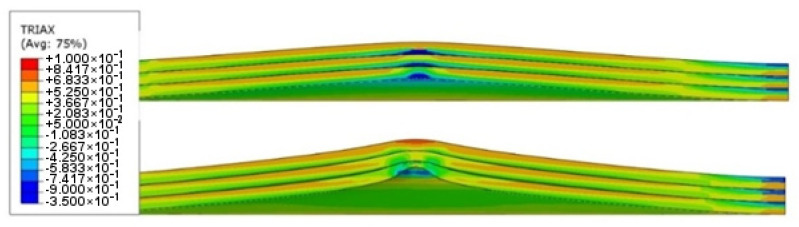
Stress triaxiality visualization in section view during the puncture of three titanium alloy sheets.

Analogous simulations were performed on three layers of Ti6Al4V titanium alloy sheet. The results of this simulation are also presented in the form of visualization of HMH stress, PEEQ strain, temperature increase, and stress triaxiality in individual layers. The maximum values in this simulation were an HMH stress of 1263 MPa, a PEEQ of 0.5306, and a sheet temperature increase of 393 K. All visualizations are shown in Figure 26, Figure 27, Figure 28 and Figure 29. It can be observed that the temperature change can affect some mechanical parameters of the considered titanium alloy [28].

**Figure 30:**
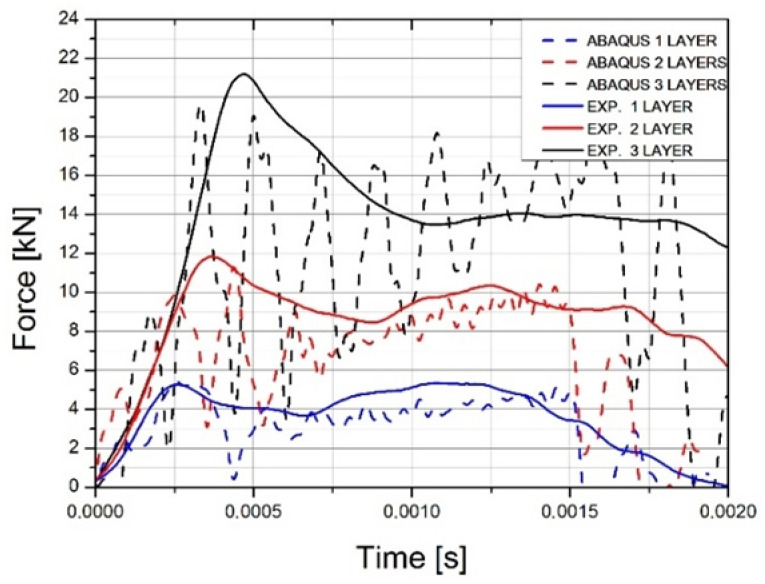
Comparison of force at damage initiation for carried out experiments and simulations.

In the next step, the forces obtained from the INSTRON machine were compared with the results of the FE simulation, which made it possible to compare the accuracy of the applied material model. The results of the analysis were filtered using the Butterworth filter (available in ABAQUS) at a 20,000 Hz cut-off frequency (COF). The Butterworth filter is one of the most commonly used filters, with maximum flat amplitude in the passband bandwidth. The Butterworth filter was used to eliminate numerical noise. The COF, set at 20 kHz, was selected over the course of subsequent analyses using COF values from 5 to 30 kHz. The selected frequency had the least interference with the achieved maximum values and with the waveform. The approximate maximum forces at the initiation of damage were obtained. For a single plate experiment, the force was 5.33 kN, while the simulation obtained 5.41 kN; for two layers, the maximum force was 11.85 kN for the experiment and 11.32 kN for the simulation. Force values for three layers were found as 21.2 kN for the experiment and 19.7 kN for the simulation. Force diagrams are shown in Figure 30, which is a comparison of all obtained results related to force at damage initiation. The difference in results might be due to a too simple friction model between the striker and the sheet, incorrect selection of coefficient of friction, the approximate fracture locus, or the imperfection of the constitutive model. Since the titanium alloy was supplied in the form of a rolled sheet, it is characterized by an anisotropy that was not included in the calculation.

The use of high-impact velocities caused difficulties in modeling the observed phenomenon, which exhibited a rapidly varying character. The obtained calculation results have an oscillatory character, resulting from the specificity of the numerical model and requiring filtering.

**Figure 31:**
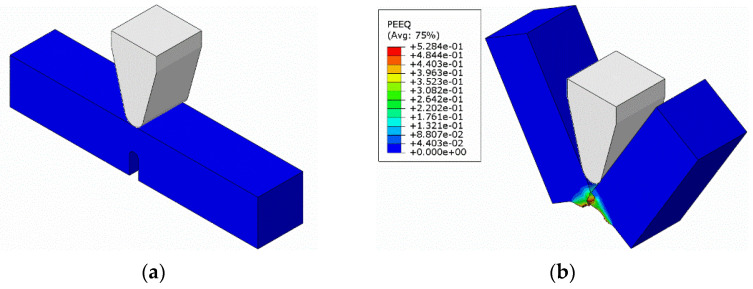
View of the impact strength simulation: (**a**) before impact; (**b**) after impact.

Due to the fact that the material used for experimental research was only in the form of metal sheets, the standard impact strength for the Charpy standard U-notched specimen was determined in the simulation using the JC model. The velocity of the striker was given a value of 12.3 m/s, as in the simulation above. The impact strength was *KC* = 122 J/cm^2^. The model of this simulation is shown in Figure 31.

## 4. Conclusions

The application of a drop-weight tester equipped with a conical striker allowed evaluation of the energy-absorbing properties of Ti6al4V titanium alloy sheets and allowed us to analyze phenomena occurring during the initiation and development of material damage. The study and analysis of the energy-absorbing properties of the titanium alloy Ti6Al4V layer structure under dynamic impact loading using a drop-weight impact tester revealed that:

- The character of the resulting damage takes the form of petaling; a similar mechanism was achieved by simulation.
- The maximum values of absorbed energy are 84 J for a single plate, 205 J for two plates, and 325 J for three plates.
- The absorbed energy increase during the puncture of two or three layers of titanium sheets is not an exact multiple of the energy measured for a single sheet. It is probable the cause of the discrepancy is the friction between the layers of material.
- The temperature values obtained from the puncture simulation of the titanium alloy samples are 287.3 K for one layer, 385.7 K for two layers, and 393 K for three layers. A slight difference in the maximum temperature between two and three layers can be seen. Despite this, the value continues to rise.
- The maximum force values for damage initiation are 5.33 kN for a single plate, 11.85 kN for two plates, and 21.2 kN for three plates.

The study of simple multi-layer structures of Ti6Al4V titanium alloy consisting of sheet metal is an introduction to the behavior of polymer-titanium and polymeric-glass-titanium composites. In further analyses, titanium alloy glue connection can be tested, using reinforcement in the form of glass or carbon fiber.

## Figures and Tables

**Table 1:** Mechanical properties of Ti6Al4V [21].

Mechanical Properties of Titanium Alloy Ti6Al4V	
Tensile strength, *Rm* (MPa)	950
Yield stress, *Re* (MPa)	880
Elongation, *A* (%)	14
Hardness (HB)	334
Shear modulus (GPa)	44
Young’s modulus (GPa)	115
Poisson’s ratio	0.342
Tensile strength, *Rm* (MPa)	950

**Table 2:** Johnson Cook constitutive model coefficients.

Gauge Length	*A* (MPa)	*B* (MPa)	*N* (-)	*C* (-)	*m* (-)	*ε*_0_ (s^−1^)	*T_r_* (K)	*T_m_* (K)
18 mm	938.67	714.68	0.69	0.00916	0.77	10^−4^	296	1923
30 mm	953.81	841.36	0.78	0.00872	0.77	10^−4^	296	1923
av	946.24	778.02	0.735	0.00894	0.77	10^−4^	296	1923

## Data Availability

Not applicable.
